# Quantitation of ergosterol content and gene expression profile of *ERG11* gene in fluconazole-resistant *Candida albicans*

**DOI:** 10.29252/cmm.3.1.13

**Published:** 2017-03

**Authors:** F Alizadeh, A Khodavandi, S Zalakian

**Affiliations:** 1 Department of Microbiology, Yasooj Branch, Islamic Azad University, Yasooj, Iran; 2 Department of Biology, Gachsaran Branch, Islamic Azad University, Gachsaran, Iran

**Keywords:** *Candida* species, *ERG11*, Fluconazole, Resistant

## Abstract

**Background and Purpose:**

The frequency of opportunistic fungal infections in immunocompromised patients*, *especially by* Candida *species*,* has sharply increased in the last few decades. The objective of this study was to analyse the ergosterol content and gene expression profiling of clinical isolates of fluconazole-resistant *Candida albicans.*

**Materials and Methods:**

Sixty clinical samples were identified and collected from immunocompromised patients, namely recurrent oral, vaginal, and cutaneous candidiasis, during 2015-16. Antifungal susceptibility testing of fluconazole against clinical *Candida* species was performed according to Clinical and Laboratory Standards Institute guidelines. Ergosterol content and gene expression profiling of sterol 14α-demethylase (*ERG11*) gene in fluconazole-susceptible and –resistant *C. albicans* were investigated.

**Results:**

The specimens consisted of *C. albicans* (46.67%), *Candida krusei* (41.67%), and *Candida tropicalis *(11.67%). All the isolates were resistant to fluconazole. No significant reduction was noted in total cellular ergosterol content in comparison with untreated controls in terms of fluconazole-resistant *C. albicans.* The expressionlevel of *ERG11* gene was down-regulated in fluconazole-susceptible *C. albicans.* Eventually, the expression pattern of *ERG11 *gene revealed no significant changes in fluconazole-resistant isolates compared to untreated controls. The results revealed no significant differences between fluconazole-susceptible and –resistant *C. albicans* sequences by comparison with *ERG11* reference sequence.

**Conclusion:**

Our findings provide an insight into the mechanism of fluconazole resistance in *C. albicans*. The mechanisms proposed for clinical isolates of fluconazole-resistant *C. albicans* are alteration in sterol biosynthesis, analysis of expression level of *ERG11* gene, and analysis of gene sequences. Nonetheless, further studies are imperative to find molecular mechanisms that could be targeted to control fluconazole resistance.

## Introduction

The incidence of resistance to *Candida* treatment has surged during the recent decades. Emergence of fluconazole-resistant *Candida* species has been progressively reported in the last 30 years [1]. Several major mechanisms leading to azole resistance have been elucidated in pathogenic fungi such as *Candida albicans* [2-6]. Declined effective drug concentrations can be achieved by overexpression of a drug’s molecular target, which gives rise to drug resistance. Changes in sterol 14α-demethylase (*ERG11*), the target of azole antifungals, are associated with azole resistance in *C. albicans* [6-9]. 

The regulation of the *ERG* genes and proteins that mediate fungal ergosterol biosynthesis remains unresolved in azole-resistant isolates. Data suggests that azole activity is directed against lanosterol 14-α-demethylase (Erg11p), which is involved in the biosynthesis of ergosterol. 

*Erg11p* is a cytochrome P450 enzyme from family 51 (CYP51) encoded by the *ERG11* gene [10, 11]. This enzyme converts lanosterol to ergosterol, which catalyses the oxidative removal of the 14α-methyl group from lanosterol. The sterol 14-α-demethylase contains a heme moiety in its active site. The unhindered nitrogen of the azoles binds to the heme iron of *Erg11p, *thus, inhibiting enzymatic reaction*.* In addition, a second nitrogen in the azoles has the potential to interact directly with the apoprotein of lanosterol-demethylase. The inhibition of *Erg11p* leads to the accumulation of 14α-methylated sterols, thereby blocking the biosynthesis of ergosterol and leading to defects in membrane and cellular integrity [5, 12].

Resistance to azole antifungal agents has been associated with *ERG11* gene expression changes and/or point mutations and alterations in the ergosterol biosynthetic pathway. Overexpression of *ERG11* increases the *Erg11p* enzyme copy number and results in elevated ergosterol synthesis, which overwhelms the capacity of the antifungal drug. The effect of *ERG11* gene alterations on antifungal susceptibility was described by several studies on*C. albicans* [5, 8, 9, 13]. In the present study, we performed ergosterol content and gene expression profiling and gene sequences analysis of fluconazole-susceptible and -resistant *C. albicans* isolates.

## Materials and Methods

***Isolates and growth conditions***

The reference strains of *C. albicans* (ATCC 14053)*, C. krusei* (ATCC 6258), and *C. tropicalis* (ATCC 750) were used. Sixty isolates of *Candida* species were obtained from randomly selected immunocompromised patients with recurrent oral (n=24), vaginal (n=22), and cutaneous (n=14) candidiasis in Omidiyeh, Khuzestan, Iran, during April 2015-April 2016. All the isolates were stored in sterile distilled water. The clinical isolates were identified on the basis of their morphological, biochemical, and molecular characteristics such as macromorphology on Sabouraud Dextrose Agar (SDA; Difco Laboratories, US), colony color on CHROMagar *Candida* medium (CHROMagar Company, France), germ tube formation, carbohydrate and nitrate assimilations, carbohydrate fermentation, urease test, and molecular methods. 

Carbohydrate assimilation test was carried out using Yeast Nitrogen Base (YNB) medium (Difco, US) as described by Evans and Richardson [14] with slight modifications. Briefly, 100 μl of the fresh cell suspension (1-5 × 10^6^ yeast cells/ml) was poured on YNB medium and kept at room temperature for 15 min to dry. Then, 50 μl of the prepared 8% (w/v) carbohydrate (glucose, maltose, sucrose, lactose, trehalose, xylose, or starch) was added to paper discs and placed on the agar surface. The plate was incubated at 30°C for 48-72 h and observed for visible growth around the disc.

The nitrate assimilation study was carried out with Yeast Carbon Base (YCB) medium (Difco, US) and discs containing KNO_3_. Carbohydrate fermentation medium containing peptone (1.5%), sodium chloride (0.5%) with Andrade indicator was prepared and poured into tubes containing inverted Durham tube and autoclaved at 121ºC for 15 min. Filter sterilized of the desired carbohydrate (glucose, maltose, sucrose, lactose, or trehalose) was added at the concentration of 2% to the medium and inoculated with 100 μl of the fresh cell suspension (1-5 × 10^6^ yeast cells/ml). The tubes were incubated at 30°C for 48-72 h and examined for the production of acid (pink color) and gas (in Durham tube). 

Urease test was performed in accordance with the manufacturer's instructions (Quelab, UK) with slight modifications. The urea agar base was dissolved in water and autoclaved at 121ºC for 15 min. Filter sterilized of the 10X concentrated urea was added to cooled urea agar base. Then, it was mixed gently and incubated at 25ºC for up to 2-5 days with loosened caps. Polymerase chain reaction (PCR) was performed using the universal fungal primers ITS1 (5'- TCC GTA GGT GAA CCT GCG G-3') and ITS4 (5'- TCC TCC GCT TAT TGA TAT GC-3'). The identity of the PCR products was confirmed by DNA sequencing method. The sequence similarity was analyzed via nucleotide BLAST software. *Candida* species were plated onto SDA containing 300 μg/ml of chloramphenicol at 35–37°C for 24 h to ensure viability and purity prior to testing [14-15]. 

***In vitro susceptibility testing***

Minimum inhibitory concentrations (MICs) were assayed, according to recommendations provided by the Clinical and Laboratory Standards Institute (CLSI) M27-A3. The test was carried out using 96-well U-bottom tissue culture microplates containing 100 µl/well of the two-fold dilution of the different concentrations of fluconazole (range: 0.03-64 μg/ml) in RPMI-1640 with L-glutamine (Sigma-Aldrich, US) buffered to pH 7.0 with 0.165 M morpholino-phosphonyl sulfate (MOPS). Yeast cell inocula were prepared (530 nm, abs 0.08–0.1) and diluted to reach a final concentration of 0.5 × 10^3^–5 × 10^3^ yeast cells/ml in the wells. Thereafter, the plates were incubated at 35°C and MICs were measured at 530 nm from two independent experiments in three separate technical replicates using a Stat Fax 303 Reader (Awareness Technology, Inc., US) after 24 h. The MIC_90_ was defined as the lowest concentration of the antifungal agent that caused 90% growth inhibition compared to control growth. The MIC breakpoints for ﬂuconazole were determined as susceptible (MIC ≤ 2 μg/ml), dose dependent (MIC = 4 μg/ml), and resistant (MIC ≥ 8 μg/ml) [16-19].

***Ergosterol quantification***

*C. albicans* ATCC 14053 (which is susceptible to fluconazole) and *C. albicans* isolate (which is resistant to fluconazole; cell mass: 25 mg) were added to fluconazole at different concentrations based on MIC (2 × MIC, 1 × MIC, ½ × MIC, and ¼ × MIC) and incubated at 35°C for 24 h. The *C. albicans* cells were harvested by centrifugation at 1,643 *g* for 5 min at 4°C and washed. The net wet weight of the cell pellet was determined. For the extraction of lipids, 3 ml of an ethanolic solution of 25% potassium hydroxide was added to each cell mass and incubated at 85°C for 1 h in a water bath. 

The tubes were further cooled to room temperature. Lipids were then extracted by addition of a mixture of 1 ml of sterile distilled water and 3 ml of *n*-heptane (Sigma-Aldrich, US) followed by vigorous vortex mixing for 3 min. The supernatant was removed, and the reading was performed in spectrophotometer at 282 and 230 nm. A calibration curve with standard ergosterol was constructed and used to calculate the amount of ergosterol. The results were expressed as the percentage of ergosterol in comparison with the untreated controls [20]. 

***Semi quantitative reverse transcriptase-(RT) PCR***

***RNA preparation***

A suspension containing different concentrations of fluconazole based on MIC (2 × MIC, 1 × MIC, ½ × MIC, and ¼ × MIC) and 1×10^6^ cells/ml of fluconazole-susceptible and -resistant *C. albicans* were prepared. The mixture was centrifuged for 10 min at 3000* rpm* and washed using phosphate-buffered saline (PBS) at least three times. Total RNA was isolated using the RNeasy Mini Kit (Qiagen, Germany) in accordance with the manufacturer's instructions with slight modifications. RNA quality and quantity were verified both electrophoretically and using a NanoDrop ND-1000 Spectrophotometer (NanoDrop Technologies Inc., DE). To avoid DNA contamination, the RNA samples were treated with RNAse-free DNase I (Fermentas, US).

***cDNA amplification***

First-strand cDNA was synthesized from 0.5 µg total RNA using the Moloney-Murine Leukemia Virus (MMLV) reverse transcriptase and random hexamer oligonucleotides (Fermentas, US) according to the manufacturer's instructions. 

***RT-PCR***

*C. albicans* *ERG11* gene was amplified from the synthesized cDNA with primers as described in Table 1. Moreover, actin (*ACT*) was established as a house-keeping gene to normalize the dissimilar RNA concentrations during RNA extraction. The RT reactions were performed in triplicate at 95°C for 4 min, followed by 26 cycles of three-step cycling, denaturation at 94°C for 40 s, annealing at 56°C for 45 s, extension at 72°C for 45 s, and a final extension at 72°C for 10 min in a TPersonal Thermocycler (Biometra, Germany) [16]. 

***RT-PCR data analysis***

The gene expression values from AlphaImager HP imaging system were generated by volume-based analyses using the standard volumes and regression curve with logistic regression method. Gel calibration was performed using Norgen’s quantitative DNA molecular weight ladders (Application Note 8). The concentration of PCR products amplified from the target and reference genes was quantitated by comparing to known standard volumes (concentration of DNA mass standard [MassRuler Low Range DNA Ladder, Ready-to-Use, Fermentas]) according to manufacturer’s operating instructions of AlphaImager software. 

The relative expression ratio was calculated by the conventional method based on concentration of PCR products as follows: fold change in target gene expression = target/reference ratio in experimental sample relative to target/reference ratio in untreated control sample. Gene with statistically significant (*P≤0.05*) variation and fold changes of ≥ 2-fold and ≤ 0.5 were classified as significantly up-regulated and down-regulated, respectively. The PCR products in the agar were excised and purified using the QIAquick Gel Extraction kit (Qiagen, US). DNA sequences obtained from the QIAquick Gel Extraction kit were confirmed by DNA sequencing (First BASE Laboratories Sdn. Bhd., Malaysia) and verified via NCBI database [16]. Sequences were analysed using SeqScape software (Applied Biosystems, US) by comparison with *ERG11* reference sequence (GenBank accession number GQ202068).

***Statistical analysis***

Statistical analysis was performed using SPSS, version 20.0. The obtained data was expressed as mean±standard error. *P-value* less than 0.05 was considered statistically signiﬁcant. 

## Results

In general, 46.67%, 41.67%, and 11.67% of the clinical yeast isolates were identified as *C*. *albicans*,*C. krusei*, and *C. tropicalis*, respectively. The most predominantly isolated species was *C. albicans*. 

***In vitro susceptibility testing***

The ﬂuconazole susceptibility results for *Candida *isolates are presented in Table 2. According to the interpretive criteria for ﬂuconazole susceptibility, all the 60 (100%) *Candida* isolates were resistant to ﬂuconazole in immunocompromised patients. Table 3 illustrates the MIC_90 _distribution (μg/ml) of *Candida* isolates against fluconazole.

***Quantitation of Candida albicans ergosterol content***

**Figure 1:**
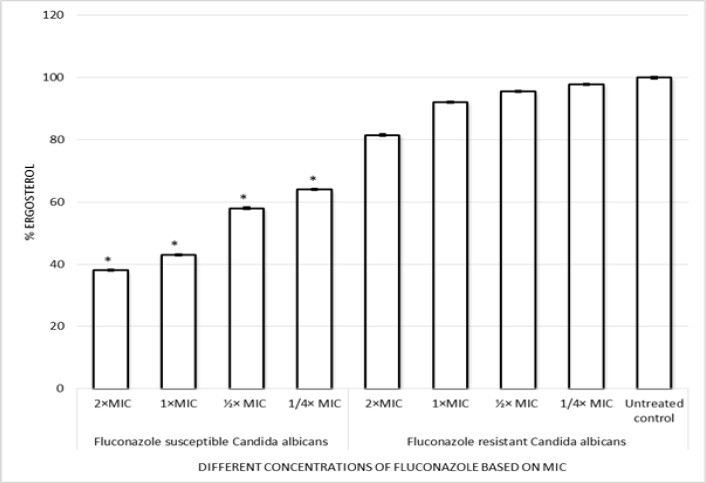
Percent ergosterol levels of fluconazole-susceptible and -resistant *C. albicans *after 24 h of treatment with different concentrations of fluconazole based on MIC (2 × MIC, 1 × MIC, ½ × MIC, and ¼ × MIC). Data are means with standard error from three independent experiments in triplicate assays. Statistically significant differences between the treatment isolates and controls are indicated with an asterisk (*P≤0.05*).

Figure 1 demonstrates the effect of fluconazole on ergosterol content in fluconazole-susceptible and –resistant *C. albicans*. The total ergosterol content was determined for each isolate grown in different concentrations based on MIC (2 × MIC, 1 × MIC, ½ × MIC, and ¼ × MIC) of fluconazole. No significant differences in the mean amount of ergosterol produced by *C. albicans* grown in the absence of fluconazole were observed regardless of the degree of fluconazole susceptibility (*P>0.05*). As shown in Figure 1, the mean reduction in total cellular ergosterol content for susceptible isolates for 2 × MIC, 1 × MIC, ½ × MIC, and, ¼ × MIC of fluconazole were 62%, 57%, 42%, and 36%, respectively. In contrast, for resistant isolates, the mean decrease in total cellular ergosterol content for 2 × MIC, 1 × MIC, ½ × MIC, and ¼ × MIC of fluconazole were 18.5%, 8%, 4.5%, and 2.2%, respectively.

**Table 1:** Oligonucleotide primers used for polymerase chain reaction

**Primer**		**Orientation**	**Sequence**	**Length (bp)**	**Reference**
*ERG11*	Forward		5' TTGGTGGTGGTAGACATA 3'	163	[16]
	Reverse		5' TCTGCTGGTTCAGTAGGT 3'		
*ACT*	Forward		5' ACCGAAGCTCCAATGAATCCAAAATCC 3	516	[16]
	Reverse		5' GTTTGGTCAATACCAGCAGCTTCCAAA 3′		

**Table 2:** Fluconazole susceptibility in *Candida* isolates from immunocompromised patients

**Isolates**		**Fluconazole susceptibility**		
		**S**	**S-DD**	**R**
*C. albicans*	Number (*n*)	0	0	28
	Percentage	0	0	100
*C. krusei*	Number (*n*)	0	0	25
	Percentage	0	0	100
*C. tropicalis*	Number (*n*)	0	0	7
	Percentage	0	0	100

S: susceptible; S-DD: susceptible dose-dependent; R: resistant

***Analysis of gene expression patterns***

**Figure 2:**
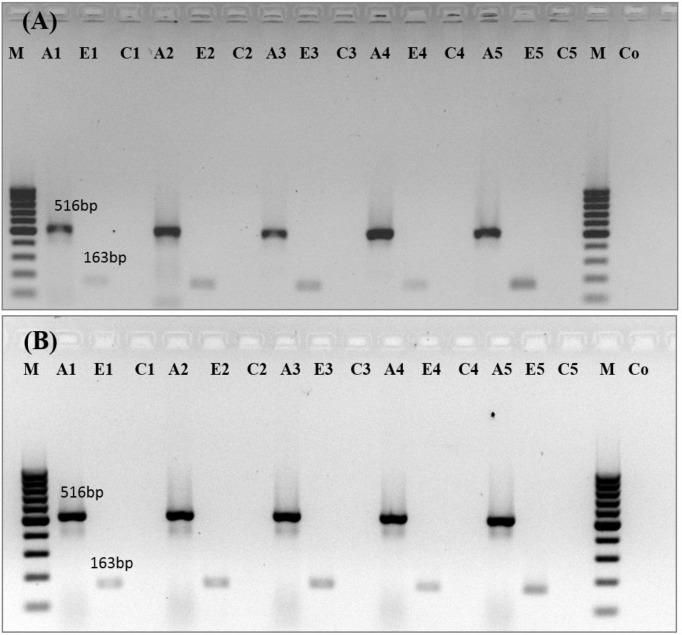
Expression analysis of *ERG11* gene in fluconazole-susceptible (A) and -resistant (B) *C. albicans *treated with different concentrations of fluconazole based on MIC (2 × MIC, 1 × MIC, ½ × MIC, and ¼ × MIC) by semi-quantitative RT-PCR. M: 100 bp DNA Ladder, A1: *Actin* with 2 × MIC concentration of fluconazole, E1: *ERG11* with 2 × MIC concentration of fluconazole, C1: Internal control without M-MuLV reverse transcriptase, A2: *Actin* with 1 × MIC concentration of fluconazole, E2: *ERG11* with 1 × MIC concentration of fluconazole, C2: Internal control without M-MuLV reverse transcriptase, A3: *Actin* with ½ × MIC concentration of fluconazole, E3: *ERG11* with ½ × MIC concentration of fluconazole, C3: Internal control without M-MuLV reverse transcriptase, A4: *Actin* with ¼ × MIC concentration of fluconazole, E4: *ERG11 *with ¼ × MIC concentration of fluconazole, C4: Internal control without M-MuLV reverse transcriptase, A5: *Actin* without fluconazole (untreated control), E5: *ERG11* without fluconazole (untreated control), C5: Internal control without M-MuLV reverse transcriptase, Co: Control negative for PCR.

**Figure 3:**
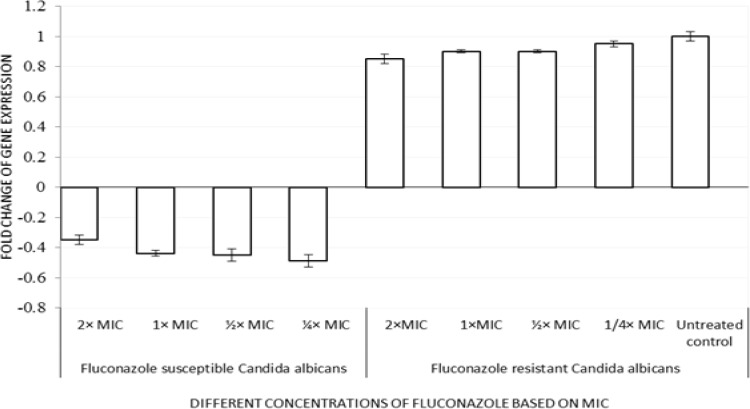
Relative quantitation of *ERG11* gene expression (normalized to house-keeping gene, actin) in fluconazole-susceptible and -resistant *C. albicans *treated with different concentrations of fluconazole based on minimum inhibitory concentration (2 × MIC, 1 × MIC, ½ × MIC, and ¼ × MIC). Data are means of fold changes with standard deviation from three independent experiments amplified in triplicate.

The expression of *ERG11 *gene in fluconazole-susceptible and -resistant *C. albicans* treated with different concentrations of fluconazole based on MIC (2 × MIC, 1 × MIC, ½ × MIC, and ¼ × MIC) is illustrated in figures 2 and 3. *ERG11* expression was significantly down-regulated (*P≤0.0001*) in the fluconazole-susceptible *C. albicans* cells treated with different concentrations of fluconazole. The mean relative *ERG11 *gene expression levels in susceptible isolates for 2 × MIC, 1 × MIC, ½ × MIC, and ¼ × MIC were 0.35±0.03, 0.44±0.02, 0.45±0.01, and 0.49±0.01, respectively. However, no significant changes were observed in fluconazole-resistant isolates in comparison with untreated controls.

The sequences displayed high similarity via nucleotide BLAST in Gene Bank and confirmed homology to the related genes. No differences were observed in fluconazole-susceptible and –resistant *C. albicans* by comparison with *ERG11* reference sequence.

**Table 3:** MIC_90 _distribution (μg/ml) of 60 *Candida *isolates against fluconazole

**Isolates**	**MIC**_90_ ** distribution (μg/ml)**												
	**0.03**	**0.06**	**0.125**	**0.25**	**0.5**	**1**	**2**	**4**	**8**	**16**	**32**	**64**	
	**n (%)**												
*C. albicans*	0	0	0	0	0	0	0	0	0	1 (3.57)	11 (39.29)	16 (57.14)
*C. krusei*	0	0	0	0	0	0	0	0	0	3 (12)	12 (48)	10 (40)
*C.* *tropicalis*	0	0	0	0	0	0	0	0	0	0	4 (57.14)	3 (42.86)

## Discussion

The incidence rate of *Candida* infections has grown during the recent decades, and the spreading problem of antifungal drug resistance in the recent years has magnified the need for improved antifungal susceptibility tests [21-23]. In this study, we provided data on antifungal susceptibility of *Candida* isolates, and all the 60 isolates from immunocompromised patients were resistant to fluconazole. Fluconazole is still considered the first-line treatment for *Candida*  infections because it offers the advantages of both oral and parenteral formulations, favorable bioavailability, and low level of toxicity [24, 25]. The fluconazole resistance of *Candida* species has been increasingly reported in the last 30 years [1]. 

Antifungal azoles such as fluconazole target lanosterol 14α-demethylase, which is a product of the *ERG11* gene. Multiple molecular mechanisms may operate simultaneously in fluconazole resistance in *C. albicans*. These mechanisms include reduced intracellular drug accumulation, alterations in the sterol biosynthesis pathway, overexpression of the *ERG11* gene encoding the drug target enzyme 14α-demethylase, and mutations in the *ERG11* gene, which result in reduced affinity of 14α-demethylase to fluconazole and is correlated with the overexpression of membrane transport proteins [24, 26].

Our findings demonstrated no significant diminution in total cellular ergosterol content by comparison with untreated controls for fluconazole-resistant *C. albicans. *However, a significant decrease in total cellular ergosterol content was observed for fluconazole-susceptible *C. albicans.* Ergosterol is an important component of the fungal cell membrane that regulates membrane fluidity and permeability [27, 28]. In the literature, fluconazole-susceptible isolates showed a mean reduction of 72% in ergosterol content after exposure to 1 μg of fluconazole/ml, an 84% reduction after exposure to 4 μg/ml, as well as 95% and 100% reductions after exposure to 16 and 64 μg of fluconazole/ml, respectively. In contrast, fluconazole-resistant isolates showed mean reductions in ergosterol content of only 25%, 38%, 53%, and 84% after exposure to the same concentrations of fluconazole [29]. 

In addition, the expression pattern of *ERG11 *gene in fluconazole-susceptible and –resistant *C. albicans *revealed no significant changes in fluconazole-resistant isolates as compared to untreated controls. On the contrary, the expression of *ERG11* was down-regulated in the fluconazole-susceptible *C. albicans.* Our results are in partial agreement with those of Salari et al. [30], who demonstrated no significant enhancement in mRNA levels of *ERG11* in fluconazole-resistant *C. albicans. *

There were no significant differences in fluconazole-susceptible and -resistant *C. albicans* sequences by comparison with *ERG11* reference sequence. Young et al. [31] analyzed mechanisms contributing to amphotericin B resistance in *Candida lusitaniae*. The ergosterol content and expression levels of ergosterol biosynthetic genes were analyzed in multiple clinical isolates*. C. lusitaniae* amphotericin B-resistant isolates were found to have reduced ergosterol content as well as increased levels of *ERG6 *gene. Nonetheless, the expression of the *ERG3* gene was reduced in amphotericin B-resistant isolates. Their results revealed that altered expression of ergosterol biosynthetic genes could result in resistance to amphotericin B in *C. lusitaniae*.

Borecká-Melkusová et al. [32] found low or moderate expression of the *ERG11* gene, which was regulated by fluconazole addition more so than by biofilm formation. Very low or nondetectable expression of *ERG1*, *ERG7*, and *ERG25* genes was detected in *C. albicans*. The expression levels of the *ERG9* increased in the presence of fluconazole in some isolates. 

## Conclusion

Our findings provide insight into the mechanism of action of fluconazole-resistant *C. albicans*. The mechanisms proposed for clinical isolates of fluconazole-resistant *C. albicans* are alteration in sterol biosynthesis, analysis of expression level of *ERG11* gene, and alanysis of gene sequences, which deserves further investigation. Further studies are required to explore the molecular mechanisms that could be targeted to control fluconazole resistance. 
